# Research on motion characterization of goose neck in narrow space

**DOI:** 10.3389/fvets.2024.1423453

**Published:** 2024-11-20

**Authors:** Fu Zhang, Haoxuan Sun, Jiajia Wang, Xinyue Wang, Yubo Qiu, Xiahua Cui, Shaukat Ali

**Affiliations:** ^1^College of Agricultural Equipment Engineering, Henan University of Science and Technology, Luoyang, China; ^2^Longmen Laboratory, Luoyang, China; ^3^College of Engineering and Technology, Jilin Agricultural University, Changchun, China; ^4^College of Biological and Agricultural Engineering, Jilin University, Changchun, China; ^5^Wah Engineering College, University of Wah, Wah Cantt, Pakistan

**Keywords:** bionics, goose neck, narrow space, motion trajectory, joint angle

## Abstract

**Introduction:**

Inspired by the obstacle avoidance mechanism of goose neck, a theoretical design method of bionic robotic arm was proposed to solve the contradiction between high flexibility and strong bearing capacity in narrow space.

**Methods:**

Taking the goose neck as the test object, a narrow space test environment with a width of 10 cm was built, and a 6 × 4 obstacle matrix was set up, to analyze the maximum value of joint angle, motion rate and trajectory in different target areas.

**Results:**

The test results showed that the goose neck movement has continuity and transmissibility. The overall posture of the goose neck was adjusted through the synergistic movement of the anterior, middle and posterior segments to move toward the target position. The regulating effect of the anterior segment was significantly stronger than that of the middle and posterior segments. Specifically, the anterior segment of goose neck exhibited mostly transverse movement, with significant horizontal regulation; the middle segment of the goose neck was coupled with longitudinal movement, with similar movement ability in all directions, the posterior segment of the goose neck has mostly longitudinal movement, with significant height regulation.

**Conclusion:**

In addition, the YOLOv7-pose recognition network was used to recognize goose neck motion pose, which provides a new method for animal behavior research.

## 1 Introduction

Robotic arms have been used in a wide range of applications in fields such as healthcare, automotive manufacturing, aerospace, food production, and agricultural production (1–3). With the increase of industrialization level and equipment integration, a large number of jobs need to be operated in narrow space, which has high requirements for the flexibility, stability and load capacity of robotic arms (4–6). Traditional robotic arms, while having high structural rigidity and strong load capacity, lack the dexterity required for operations in narrow space. Bionic flexible robotic arms offer high flexibility and the ability to continuously deform, exhibiting greater movement and operational capabilities in narrow spaces compared with traditional articulated robotic arms. However, it is accompanied by the problem of lower load capacity. Avian neck has ultra-high motion stability with the cervical vertebra serving as the most flexible part of its spine (7). Typically, the number of vertebrae ranges from 11 to 25 (8), each capable of bending in two directions, providing flexible and stable control of the head, which can weigh several times more than the neck, to perform various activities (9). Research into the motion mechanism of the avian neck in narrow spaces can provide a theoretical basis for resolving the contradiction between the flexibility and load capacity of robotic arms.

Existing research on avian neck kinematics primarily focuses on domestic chickens, owls, ostriches, geese, etc. Movement forms include natural walking, feeding, and flying. van der Leeuw et al. (10) analyzed the characteristics and patterns of feeding and drinking movements in domestic chickens and geese, revealing that the cervical vertebrae of domestic chickens adhere to the geometric principle of maximizing angular efficiency. Furet et al. (11, 12) used CT scanning to obtain the three-dimensional surface characteristics and static maximum range of flexion of an owl's cervical vertebrae, and established a motion model imitating the bird neck linkage based on surface contact characteristics. Krings et al. (13, 14) employed X-ray technology to capture the natural neck posture when the heads of live and cadaveric owls were rotated, and utilized CT scanning to study the shape of a single vertebra. Their study indicated that rotational motion can be described as a combination of movement on the yawing axis (yawing) and the cross-rolling axis (rolling). Panyutina et al. (15) utilized CT scanning and a joint coordinate system (JCS) to investigate extreme head turns in owls, discovering that during full head turns, the maximal joint angles alternate along the neck in three planes, with maximal axial rotation (to the side of the head turn) followed by maximal sagittal bending (in the ventral direction) and then by maximal lateral bending (to the side of the head turn). Kambic et al. (16) categorized the range of motion of the avian neck into three areas: the cranial joints, predominantly in ventral flexion with high axial rotation and lateral flexion activity; the caudal joints, predominantly in dorsiflexion with low axial rotation activity and high lateral flexion activity; and the intermediate joints, variable in axial rotation activity and exhibiting low lateral flexion. Wang et al. (17, 18) studied the structural characteristics of the bird neck skeleton and static motion characteristics using CT scanning, while employing biplane X-ray techniques to study the passive stabilizing motion of the goose neck. The results indicated that the goose neck has the best passive motion stability in the sagittal plane for two directions of motion. Abourachid et al. (19, 20) combined the contact characteristics of the vertebrae to establish an intervertebral motion model suitable for birds, demonstrating that the saddle-shaped articular processes of avian vertebrae limit joint mobility, with the position and orientation of the articular processes determining vertebral mobility. The orientation of the articular eminence surfaces dictates the range of motion in dorsiflexion and lateral flexion, while the axial angle of the articular zygapophyses surfaces effectively restricts longitudinal rotation. Dzemski and Christian (21) studied the ostrich's neck and showed that it could be divided into three segments with varying flexibility, the upper portion being more flexible in the dorsal-ventral and lateral directions, the middle portion having the highest dorsal-ventral flexibility, and the bottom portion having the highest lateral flexibility. Gunji et al. (22, 23) analyzed typical ostrich neck behavior by establishing an ostrich neck dynamics model and found that ostrich neck movement involved both lever and rolling actions. He et al. (24) observed that during walking, a chicken's neck extends and contracts alternately to provide intermittent fixed stability for the head. They designed a bionic vertebrae unit combining springs and universal joints to simulate chicken cervical vertebrae, investigated the connection and motion characteristics, and found that the unit could achieve an S-shaped bionic bending configuration and successfully wind and lift objects of interest, proving that the proposed robot has excellent flexibility and application potential, and that the design method is effective.

Research has been conducted on the structural characteristics of bird neck, range of motion, and the maintenance of head stability, among other aspects. Most of the studies have used CT scanning equipment to obtain the structural characteristics of bones. Anatomical methods were used to obtain the structural characteristics of muscles. *In vivo* motion studies have used X-ray video or biplane X-ray motion analysis system to test the motion of bird neck vertebrae. The research on the structure and motion characteristics of live bird neck have mainly focused on free motion in open space, and mostly on vertebral kinematic analyses. However, the motion characteristics of the bird neck in narrow space based on the overall motion posture and muscle distribution characteristics of the bird neck are still unclear, and the research to analyze its obstacle avoidance mechanism in depth has not been reported. In this research, the goose neck was selected as the subject of investigation, muscle distribution characteristics were analyzed by using goose neck MRI data. The high-speed camera system was used to collect the movement data of the goose neck in the narrow space, and the goose neck motion pose was extracted by YOLOv7-pose recognition network. Then, combining the characteristics of goose neck muscle distribution and movement posture to analyze the angular changes and movement trajectories of goose neck joints. Finally, the movement mechanism of the goose neck was explored in the narrow space, which provide the theoretical basis for the research on goose neck kinematics and design optimization of the bionic multi-joint robotic arms.

## 2 Materials and methods

### 2.1 Experimental equipment and software

The following equipment and software were used in this research:

1) MRI scanning was accomplished by Siemens Spectra 3.0T MRI scanning system (Siemens, Munich, Germany).
2) Goose neck motion data acquisition and analysis was by Phantom Miro series M110 high-speed camera system (Vision Research Inc, New Jersey, USA), and the PCC software (Vision Research Inc, New Jersey, USA).
3) Goose neck key point dataset was produced Labelme software (Massachusetts Institute of Technology, Boston, USA).
4) The Pytorch framework was used to build the YOLOv7-pose pose recognition network, and the model was trained on a DELL Precision 7820 workstation. The hardware configuration of the workstation was as follows: CPU is Intel^®^ Xeon^®^ Silver 4210R with a main frequency of 2.4 GHz, RAM is 32 GB, GPU is NVIDIA Quadro RTX 5000, Video Memory is 16 GB, and the operating system is Windows 10 Professional. For the software platform, PyCharm 2023.2.1 was used as the IDE, CUDA 12.1 as the GPU-accelerated computing platform, Python version 3.10, and PyTorch version 2.1.0.

### 2.2 Goose neck MRI experiment

In order to investigate the effect of goose neck muscles on its movement, Magnetic Resonance Imaging (MRI) data was collected from the neck of goose to obtain the distribution of goose neck muscles. The body of the goose was cleaned before the test to ensure that there is no dirt or foreign matter on the surface of the goose's body in order to minimize the noise points of the MRI data. To prevent the goose from moving and affecting the scanning effect, the goose was injected with an appropriate amount of Zoletil 50 anesthesia drug before the MRI scanning to ensure that was in sleeping state during the test and could return to the normal state after the test. The anesthesia process was completed by a professional veterinarian.

The goose neck MRI scanning test was performed at Dongfang Hospital, Luoyang City, Henan Province, P. R. China, with Siemens Spectra 3.0T MRI scanning system. The feeding conditions of test animals are in accordance with the standard (GB14925) and comply with the requirements of animal welfare (GB/T 42011-2022). Adult gray goose with growth period of 2 years and weight of about 4 kg was selected as test subject, which was able to move neck freely and had no history of neck disease. The sleeping goose was positioned in a recumbent posture on the MRI scanning platform to ensure stable fixation on the platform to minimize movement and distortion. The neck of the goose was adjusted according to the requirements of the experiment ensuring the location of the area of interest within the scanning range.

Appropriate scanning parameters were set according to the characteristics of the goose neck as shown in Table 1. The MRI scanner was started and goose neck was scanned according to the preset parameters. The stable and accurate scanning process were ensured, and the scanning time and sequence were recorded.

**Table 1:** MRI scanning parameters.

**Plane**	**TR/ms**	**TE/ms**	**Slice thickness/mm**	**SNR**
Sagittal	1,100.00	36.00	0.80	1.00
Coronal	2,950.00	98.00	4.00	1.00
Transverse	8,550.00	95.00	3.50	1.00

**Figure 1:**
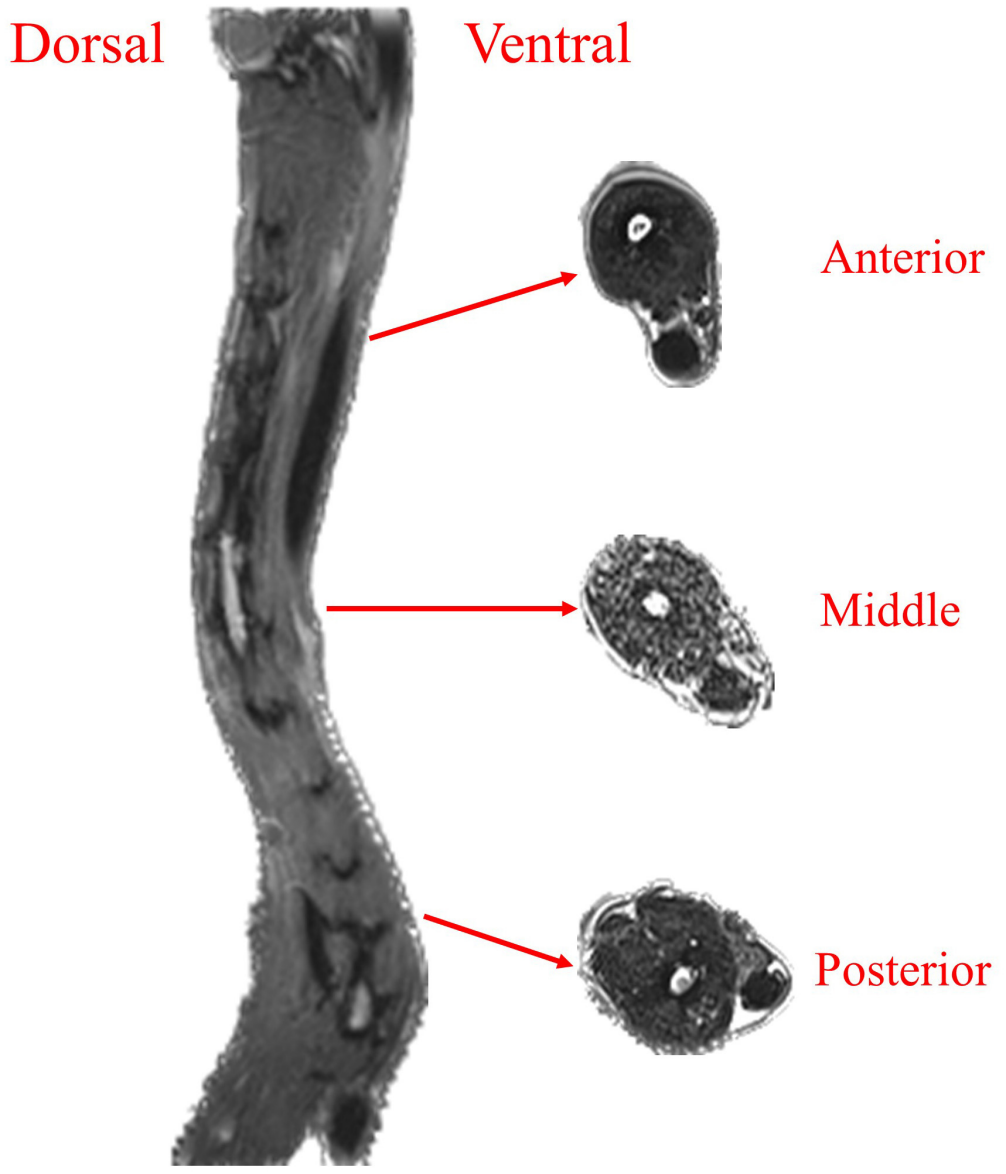
Goose neck muscle distribution. Goose neck sagittal plane muscle and transverse plane muscle distribution.

Based on the MRI scanning images, the sagittal plane, coronal plane, and transverse plane muscle distribution of the anterior, middle, and posterior segments of the goose neck were comparatively analyzed. Observing from the sagittal plane of the goose neck, it can be seen that the goose neck muscle was a long muscle that extends from the anterior to the posterior segment, as shown in Figure 1. This long muscle controlled the movement of the goose neck by means of a longitudinal connection. A similar muscular connection can be seen from the coronal plane of the goose neck in Figure 1. The muscle still showed a longitudinal distribution, connecting the anterior and posterior ends of the goose neck and controlling the lateral bending and rotational movements of the goose neck.

From the transverse plane of the goose neck, the area of the ventral muscles in the anterior segment of the goose neck was significantly larger than that of the dorsal muscles. The distribution of the muscles in the middle segment of the goose neck was relatively uniform, and there was no significant difference in the area of the ventral and dorsal muscles. The area of the ventral muscles in the posterior segment of the goose neck was significantly smaller than that of the dorsal muscles. It indicated that the ventral muscles in the anterior segment of the goose neck required greater muscle strength to support the ventral flexion movement of the anterior segment of the goose neck, the dorsal muscles in the posterior segment of the goose neck required greater muscle strength to support the dorsiflexion movement of the posterior segment of the goose neck.

### 2.3 Narrow space goose neck motion test

**Figure 2:**
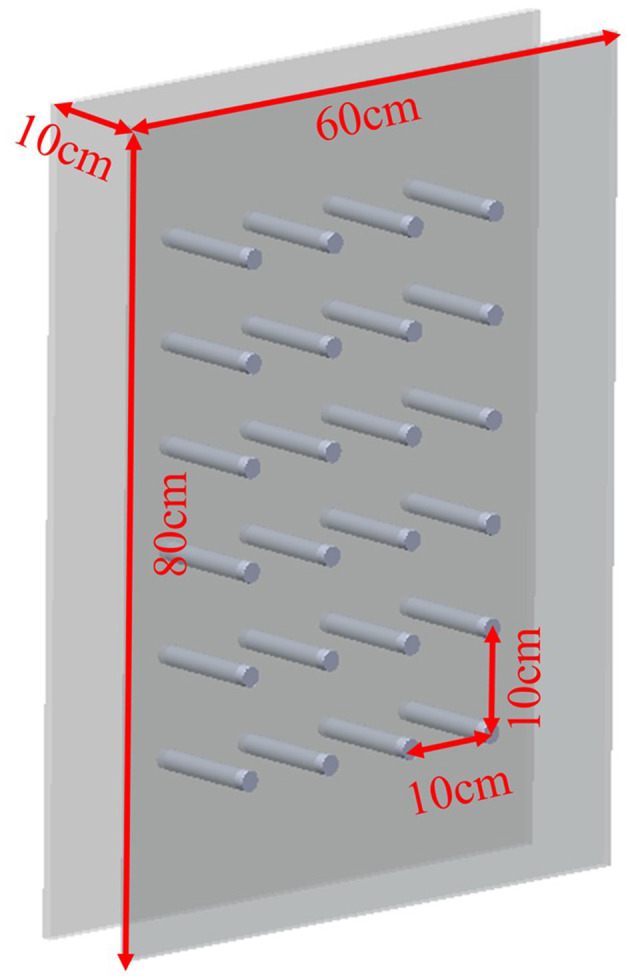
Experiment space.

To investigate the movement of a goose neck in a narrow space, it was necessary to establish a testing area containing obstacles. When constructing the test space, it was important to ensure that the space was safe and stable to ensure a reliable and accurate test. Two transparent acrylic panels were used, and arranged in parallel at 10 cm intervals to form the narrow space. Using transparent acrylic rods with a diameter of 4.5 mm, a matrix of obstacles with six rows and 4 columns was set up at 10 cm intervals in the narrow space as shown in Figure 2.

According to the distribution of goose neck muscles, the goose neck was divided into three parts, named as anterior, middle and posterior. Marking points were made at the goose cervical vertebrae C2, C5, C8, C10, and C12 with a red marker pen, labeled as L1, L2, L3, L4, and L5, respectively. The change of angle L1–L2–L3 was selected to indicate the motion of the anterior segment of the goose cervical vertebrae relative to the anterior middle segment of the cervical vertebrae. The change of angle L2–L3–L4 indicated the motion of the anterior middle segment of the goose cervical vertebrae relative to the middle segment of the cervical vertebrae. While, the change of angle L3–L4–L5 indicated the motion of the middle segment of the goose cervical vertebrae relative to the posterior segment of the cervical vertebrae. They were labeled as θ_1_, θ_2_, and θ_3_, respectively, to facilitate the subsequent analysis.

The experiments were conducted using Phantom Miro series M110 high-speed camera system for the acquisition of goose neck motion data in a confined space, and PCC software was used for the data acquisition and processing.

The motion of the goose neck exhibits uncertainty. Hence, for the ideal joint angle, motion trajectory, motion posture and other such parameters, the specific test preparation and process were carried out as follows:

1) A narrow space test environment with a width of 10 cm was built before the test.
2) In order to obtain a complete image sequence, the lens shooting direction was kept perpendicular to the direction of goose neck movement.
3) To set the high-speed camera parameters, Resolution was 1,280 × 720, Sample Rate was 200 fps, Exposure Time was 4,999.54 μs, Exposure Index was 6,400.
4) In the six rows and four columns obstacle matrix, the target position was adjusted in turn, every test was repeated and saved.

**Figure 3:**
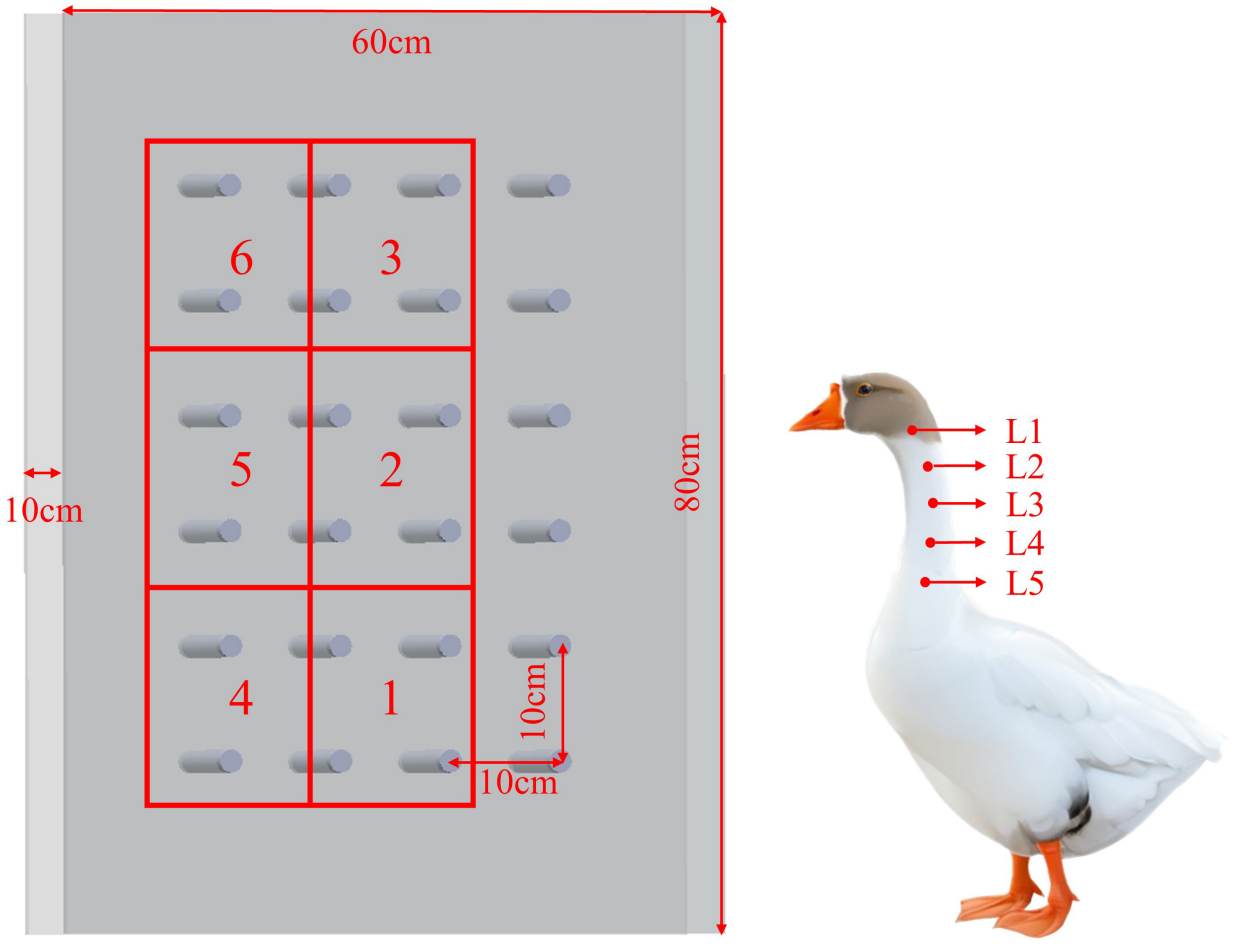
Experiment Schematic. Experimental area: Area 1, Area 2, Area 3, Area 4, Area 5, Area 6. Goose neck marker point: L1, L2, L3, L4, L5.

The experiment as shown in Figure 3.

### 2.4 Goose neck motion data processing

#### 2.4.1 Goose neck motion joint angle treatment

In order to obtain the joint angle of the goose neck movement in a narrow space, the goose neck movement data was processed by PCC software. The measurement unit parameters and the scale were set and calibrated. The distance unit was set to meters (m), the velocity unit was set to meters per second (m/s), the acceleration unit was set to meters per square second (m/s^2^), the angle unit was set to radians (rad), and the angular velocity unit was set to radians per second (rad/s). The 3-point method was used to measure the angle data of the goose neck joint angles θ_1_, θ_2_, and θ_3_, and the measurements were carried out by extracting 1 frame at every interval of 10 frames.

Goose neck kinematic joint angles θ_1_, θ_2_, and θ_3_ were processed by Matlab software. The smoothing of joint angle data was performed by six data smoothing methods, namely, moving, lowess, loess, sgolay, rlowess and rloess. The Root Mean Square Error (RMSE), Signal-to-Noise Ratio (SNR) and decidability coefficient (*R*^2^) were used as the evaluation indexes to select the optimal data smoothing method. Among these metrics, the RMSE value indicates the deviation between the denoised data and the original data, with smaller values indicating less deviation. The SNR value reflects the credibility of the data, with larger values indicating higher credibility. Lastly, the *R*^2^ value signifies the fit between the denoised data and the original data, with larger values indicating a higher degree of fit. It can be found from Table 2, that the sgolay method has the smallest RMSE value and the largest SNR and *R*^2^ values, so it was finally chosen for data smoothing.

**Table 2:** Data smoothing results.

**Norm**	**Angel**	**Moving**	**Lowess**	**Loess**	**Sgolay**	**Rlowess**	**Rloess**
RMSE	θ_1_	2.1159	2.5306	1.6042	1.1952	2.4558	1.4159
	θ_2_	1.7605	2.0548	0.9801	0.9534	2.0431	1.0528
	θ_3_	2.1113	2.6469	1.2942	0.8510	2.7277	1.1098
SNR	θ_1_	36.6728	35.1180	39.0771	41.6336	35.3788	40.1615
	θ_2_	37.7778	36.4351	42.8647	43.1049	36.4846	42.2434
	θ_3_	36.5137	34.5502	40.7647	44.4061	34.2889	42.1000
*R* ^2^	θ_1_	0.9057	0.8651	0.9458	0.9699	0.8730	0.9577
	θ_2_	0.8829	0.8406	0.9637	0.9656	0.8424	0.9581
	θ_3_	0.6438	0.4402	0.8661	0.9421	0.4055	0.9016

According to the different distances and heights of the target points, the experimental areas were divided into six distinct areas labeled as Area 1, Area 2, Area 3, Area 4, Area 5, and Area 6. The distribution of these areas is depicted in Table 3 and illustrated in Figure 3.

**Table 3:** Experimental area distribution.

**Experimental area**	**Heights/cm**	**Lengths/cm**
Area 1	15–30	20–30
Area 2	30–45	20–30
Area 3	45–60	20–30
Area 4	15–30	30–40
Area 5	30–45	30–40
Area 6	45–60	30–40

#### 2.4.2 Goose neck motion trajectory processing

**Figure 4:**
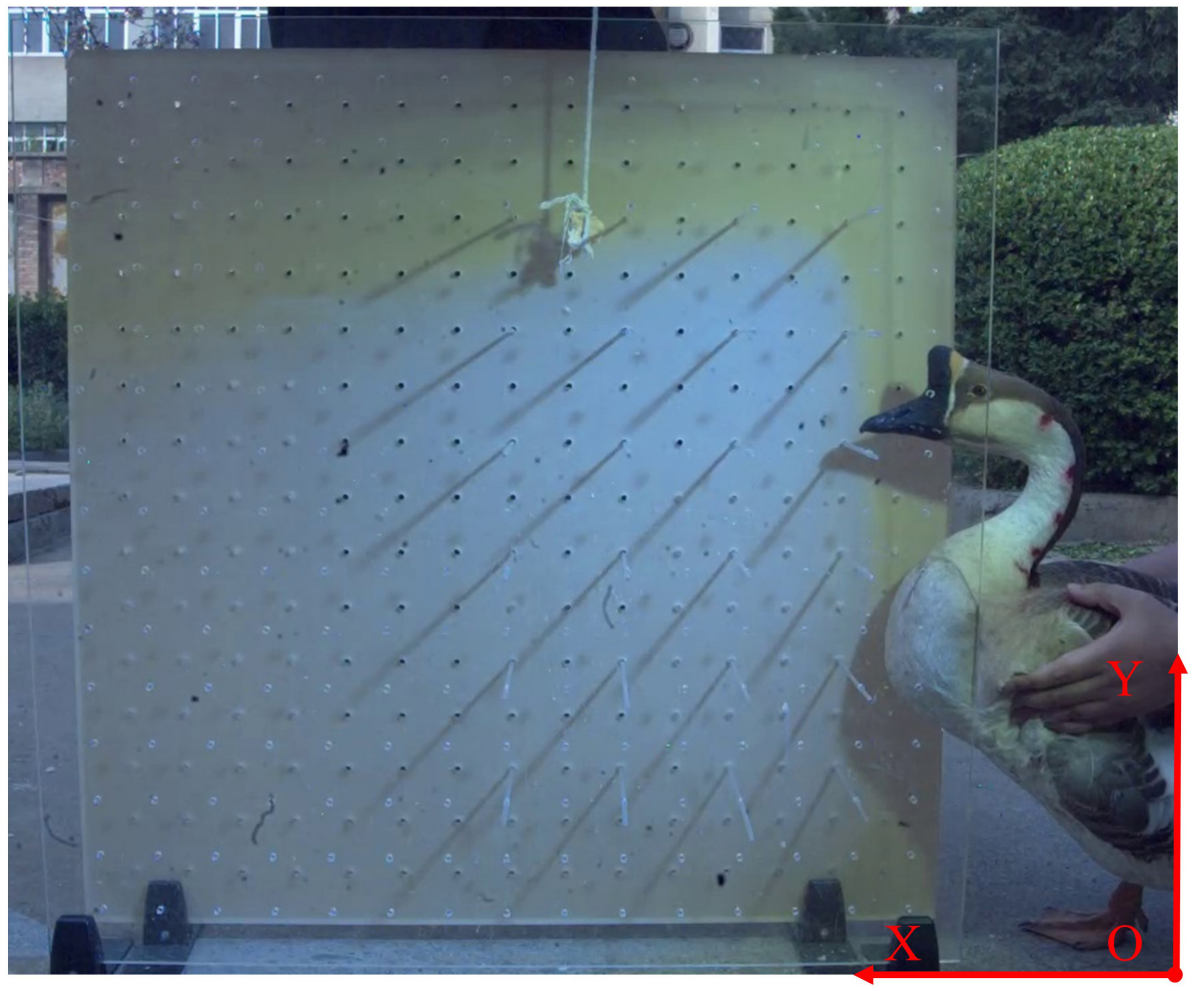
Reference coordinate system establishment.

In order to obtain the trajectory of the goose neck in a narrow space, the data collected by the high-speed camera needed to be processed to obtain the coordinates of each marking point. First, the high-speed camera system was calibrated and a reference coordinate system was established. A two-dimensional reference coordinate system was established according to the fore-and-aft and pitching motion directions of the goose neck. The lower right corner of the image area was taken as the origin of the coordinate system, and the forward and backward motion direction of the goose neck was taken as the *X*-axis, and the pitching motion direction of the goose neck was taken as the *Y*-axis. Among them, the forward movement direction of the goose neck was defined as the positive direction of *X*-axis, and the upward movement direction of the goose neck was defined as the positive direction of *Y*-axis, and the reference coordinate system as shown in Figure 4.

**Figure 5:**
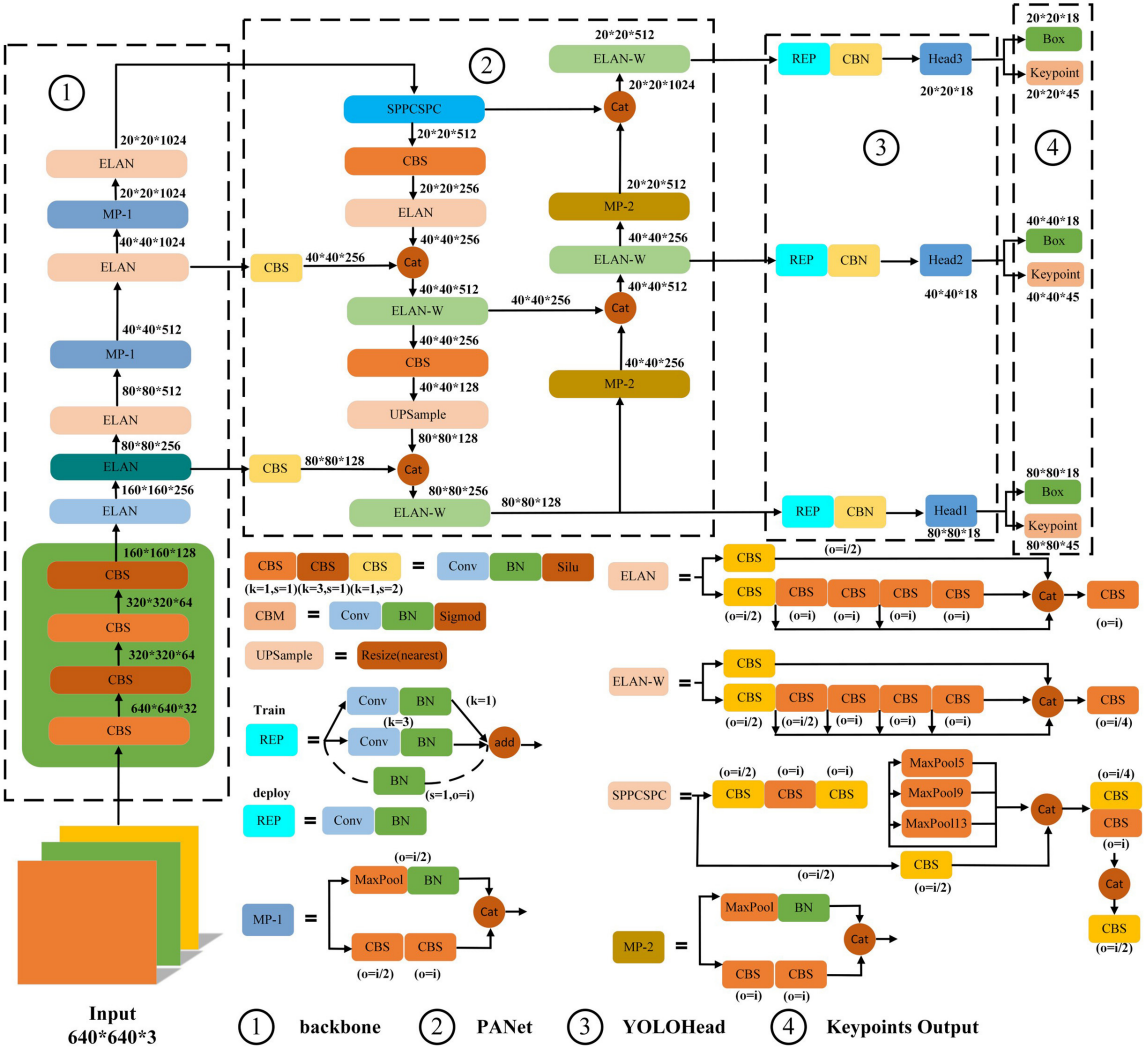
YOLOv7-pose network structure.

YOLOv7-pose recognition algorithm was used to extract the coordinate information of the marker points of the goose neck. Firstly, the ^*^.cine format video data collected through the high-speed camera system was transcoded and converted all into ^*^.mp4 format video data. Second, the ^*^.mp4 format video data were processed by using video processing algorithms to extract all the frame images. Then, in order to construct a complete dataset, frame images under different postures and lighting conditions were selected to ensure that the dataset could cover various postures and lighting conditions. Finally, the labeling tool Labelme software was used to label the selected frame images, including the whole goose neck and goose neck key point information, and generated ^*^.json type labeling files. The YOLOv7-pose network structure is shown in Figure 5.

The 2:1 ratio was used to divide the training set (3,685 images) and test set (1,806 images). Among them, the training set was used for model training and parameter optimization, and the test set was used to evaluate the model performance. In order to augment the dataset, increase the difficulty of model training and improve the model recognition accuracy, physical transformation operations were performed on the divided dataset using the following four augmentation methods; random rotation, Gaussian noise, horizontal flip, and luminance change.

The YOLOv7-pose recognition network was used, and the software platform was Pycharm 2023.2.1+CUDA 12.1+Python 3.10+torch 2.1.0. In terms of the parameter settings for the YOLOv7-pose pose recognition network, the size of the training image was set to 640 × 640 pixel, the training batch size was set to 16 and the epoch was set to 300. The optimal weights would be automatically saved after each test.

**Figure 6:**
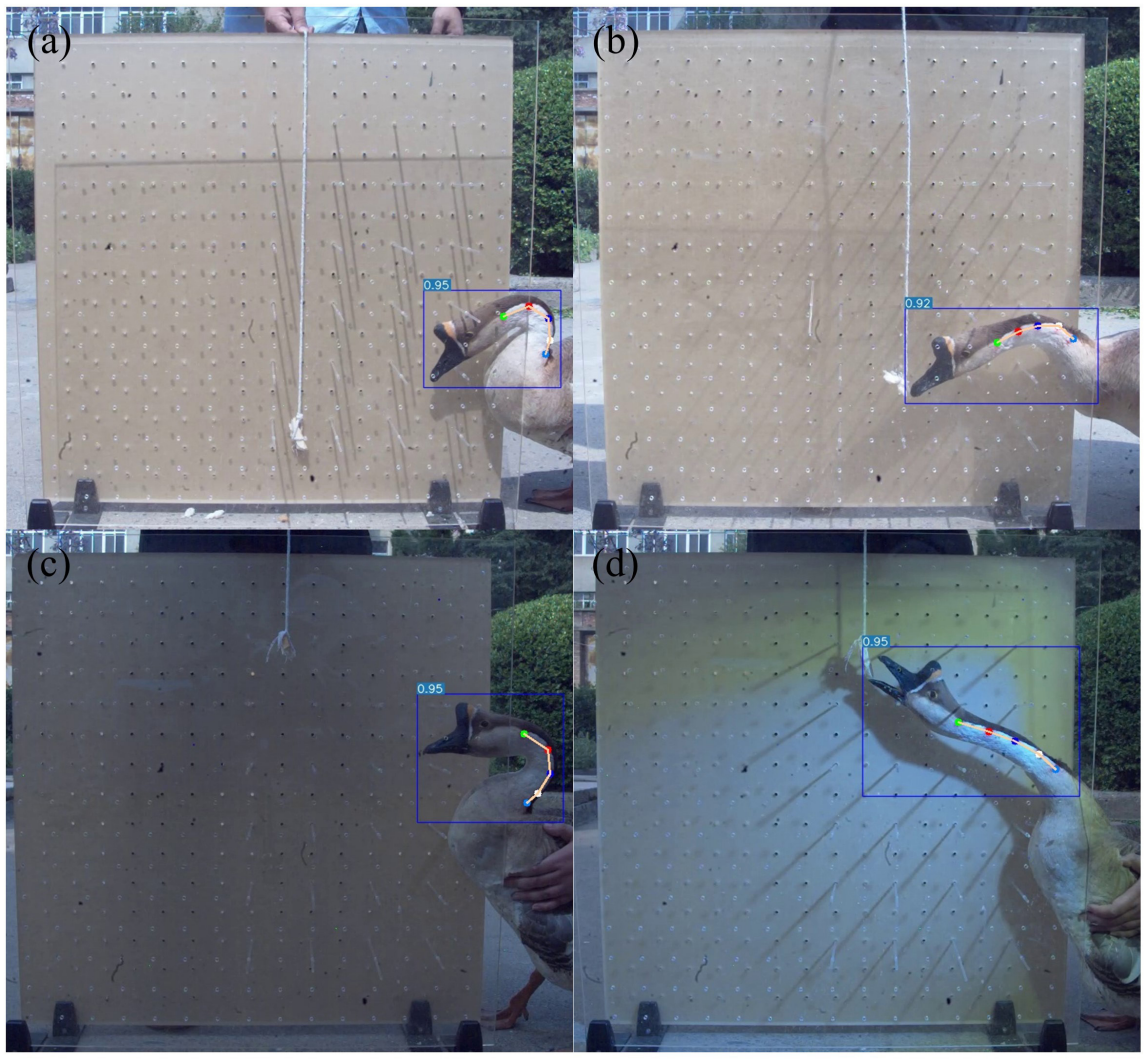
YOLOv7-pose recognition results. **(a)** Recognition in goose neck bent pose. **(b)** Recognition in goose neck extended pose. **(c)** Recognition in low light condition. **(d)** Recognition in bright light condition.

The YOLOv7-pose network was used for 300 epoch, and the AP reached 99.6% after training, with excellent training results. Based on the YOLOv7-pose pose recognition model, goose neck pose recognition was performed on all frames extracted from the high-speed camera video, as shown in Figure 6 and Table 4. The recognition results showed that the goose neck as a whole as well as the five marking points of the neck could be accurately recognized under different poses and different lighting conditions, which can be used for the analysis of the goose neck movement trajectory.

**Table 4:** Network inspection performance results.

**Precision/%**	**Recall/%**	***F*l score/%**	**Average precision/%**	**Detection speed/FPS**	**Weight size/MB**
99.4	95.1	98	99.6	36.7	153

## 3 Results

### 3.1 Goose neck motion joint angle analysis

The joint angles of the goose neck moving at different heights and distances in a narrow space were analyzed to obtain the maximum, minimum and range of the rotation angles of each joint of the goose neck, as shown in Table 5.

**Table 5:** Goose neck motion joint angles.

	**L1–L2–L3 (**θ_**1**_**)**			**L2–L3–L4 (**θ_**2**_**)**			**L3–L4–L5 (**θ_**3**_**)**		
	**MAX/**°	**MIN/**°	**VAR/**°	**MAX/**°	**MIN/**°	**VAR/**°	**MAX/**°	**MIN/**°	**VAR/**°
1	179.82	89.13	90.69	178.51	96.15	82.36	168.55	100.95	67.60
2	178.99	104.76	74.23	165.88	105.25	60.63	165.96	113.75	52.21
3	179.39	115.79	63.60	178.15	121.87	56.28	179.66	136.65	43.01
4	178.85	88.56	90.29	176.14	102.78	73.36	175.22	113.20	62.02
5	179.89	88.38	91.51	170.62	98.63	71.99	173.33	114.78	58.55
6	179.86	109.39	70.47	176.74	98.43	78.31	173.50	128.99	44.51

From the Table 5, the maximum values and rotation ranges of each joint angle of the goose neck were analyzed. The maximum rotation angle of the anterior segment θ_1_ was 179.89°, the minimum rotation angle was 88.38°, and the maximum rotation range was 91.51°. The maximum rotation angle of the middle segment θ_2_ was 178.51°, the minimum rotation angle was 96.15°, and the maximum rotation range was 82.36°. The maximum rotation angle of the posterior θ_3_ was 179.66°, the minimum rotation angle was 100.95°, and the maximum rotation range was 78.71°. The maximum rotation angle of the end segment θ_3_ was 179.66°, the minimum rotation angle was 100.95°, and the maximum rotation range was 78.71°.

**Figure 7:**
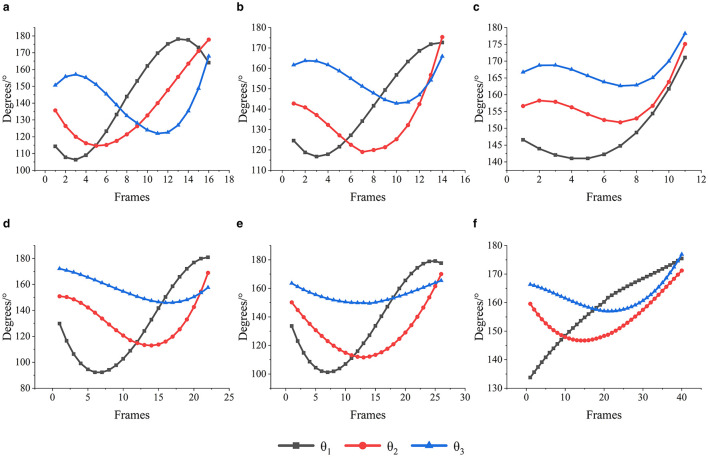
Angle of rotation of goose neck joints in different areas. **(a)** Area 1 goose neck joint rotation angle. **(b)** Area 2 goose neck joint rotation angle. **(c)** Area 3 goose neck joint rotation angle. **(d)** Area 4 goose neck joint rotation angle. **(e)** Area 5 goose neck joint rotation angle. **(f)** Area 6 goose neck joint rotation angle.

Origin software was used to analyze and process the test data of the joint angles. The rotation angles between different joints of the goose neck in each area of the narrow space are plotted in Figure 7.

As shown in Figure 7a, when the goose neck moved toward the target Area 1, the anterior segment joint angle θ_1_ firstly decreased about 10°, then gradually increased to about 180°, and finally decreased 15°; the middle segment joint angle θ_2_ firstly decreased about 20°, and then gradually increased to about 180°; and the posterior segment joint angle θ_3_ firstly increased about 10°, and then decreased about 40°, and finally increased to about 170°.

As shown in Figure 7b, when the goose neck moved toward the target Area 2, the anterior segment joint angle θ_1_ firstly decreased by about 10° and then increased to about 170°; the middle segment joint angle θ_2_ of firstly decreased by about 30° and then increased to about 175°; and the posterior segment joint angle θ_3_ of firstly decreased by about 20° and then increased to about 165°.

As shown in Figure 7c, when the goose neck moved toward the target Area 3, the anterior segment joint angle θ_1_ firstly decreased by about 10° and then increased to about 170°; the middle segment joint angle θ_2_ firstly decreased by about 10° and then increased to about 174°; and the posterior segment joint angle θ_3_ firstly decreased by about 5° and then increased to about 178°.

As shown in Figure 7d, when the goose neck moved toward the target Area 4, the anterior segment joint angle θ_1_ firstly decreased by about 40° and then increased to about 180°; the middle segment joint angle θ_2_ firstly decreased by about 40° and then increased to about 170°; and the posterior segment joint angle θ_3_ firstly decreased by about 20° and then increased to about 160°.

As shown in Figure 7e, when the goose neck moved toward the target Area 5, the anterior segment joint angle θ_1_ firstly decreased by about 55° and then increased to about 180°; the middle segment joint angle θ_2_ firstly decreased by about 40° and then increased to about 170°; and the posterior segment joint angle θ_3_ firstly decreased by about 10° and then increased to about 168°.

As shown in Figure 7f, when the goose neck moved toward the target Area 6, the anterior segment joint angle θ_1_ continued to increase to about 174°; the middle segment joint angle θ_2_ firstly decreased by about 15°, and then increased to about 170°; and the posterior segment joint angle θ_3_ firstly decreased by about 8°, and then increased to about 176°.

### 3.2 Goose neck motion trajectory analysis

**Figure 8:**
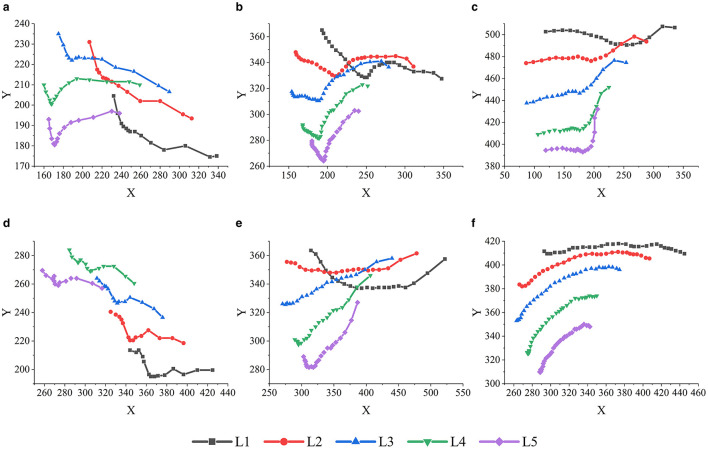
Motion trajectory of each marker points of the goose neck. **(a)** Area 1 goose neck motion trajectory. **(b)** Area 2 goose neck motion trajectory. **(c)** Area 3 goose neck motion trajectory. **(d)** Area 4 goose neck motion trajectory. **(e)** Area 5 goose neck motion trajectory. **(f)** Area 6 goose neck motion trajectory.

To further analyze the motion of the goose neck in the narrow space, the YOLOv7-pose pose recognition algorithm was used to extract the position information of the marker points L1, L2, L3, L4, and L5, and the goose neck motion trajectory is plotted in Figure 8.

As shown in Figure 8a, when the target position was in Area 1, the overall goose neck was firstly moving downward and then forward. During the forward movement of the goose neck, the marker points L1, L2, and L3 of the anterior-middle segment moved further downward, and the movement heights of the marker points L4 and L5 of the posterior segment were basically unchanged.

As shown in Figure 8b, when the target position was in Area 2, the overall goose neck was firstly moved downward, then adjusted upward, and finally moved forward. During the forward movement of the goose neck, the movement heights of the marker points L1, L2, and L3 in the anterior-middle segment were basically unchanged, and the marker points L4 and L5 in the posterior segment moved further upward.

As shown in Figure 8c, when the target position was in Area 3, the goose neck as a whole moved forward first, followed by a compound motion in the forward and upward directions. During the forward motion of the goose neck, the motion height of each marker point was basically unchanged; when the goose neck carried out the composite motion in the forward and upward directions, the forward motion magnitudes of each marker point decreased in turn, and the upward motion magnitudes increased in turn.

As shown in Figure 8d, when the target position was in Area 4, the overall goose neck moved downward first, and then forward. During the forward movement of the goose neck, the movement heights of the marker points L1 and L2 in the anterior-middle segment were basically unchanged, and the marker points L3, L4, and L5 in the middle-posterior segment moved further downward.

As shown in Figure 8e, when the target position was in Area 5, the overall goose neck firstly moved downward, then moved upward previously, and finally carried out a compound motion in the forward and upward directions. During the forward movement of the goose neck, the movement height of the marker points L1 and L2 of the anterior segment was basically unchanged, and the marker points L3, L4, and L5 of the middle-posterior segment further moved upward; when the goose neck carried out the composite movement in the forward and upward directions, the amplitude of the forward movement of each marker point decreased in turn, and the amplitude of the upward movement increased in turn.

As shown in Figure 8f, when the target position was in Area 6, the overall movement of the goose neck was upward first, followed by forward movement. During the forward movement of the goose neck, anterior segment marker point L1 movement height was basically unchanged, middle-posterior segment marker points L2, L3, L4, L5 forward movement amplitude decreased in turn, and upward movement amplitude increased in turn.

## 4 Discussion

From the most values of the angles of the goose neck joint angles θ_1_, θ_2_, and θ_3_, it was found that the maximum rotation angles of the goose neck anterior segment joint angle θ_1_, the middle segment joint angle θ_2_ and the posterior segment joint angle θ_3_ are similar. Both the maximum and the minimum rotation angles decreased in order. It indicated that when the goose neck moved in a narrow space, the anterior segment exhibited the greatest adjustment capability, followed by the middle segment, with the posterior segment demonstrating relatively weaker adjustment ability. It could be seen that the adjustment effect of the anterior segment was stronger than that of the middle and posterior segments of the goose neck, and its flexibility was also better than that of the middle and posterior segments.

From the goose neck motion joint angle analysis, the overall trend of change for each joint angle was quite similar. Primarily, the angles of goose neck anterior joint θ_1_, middle joint θ_2_, and posterior joint θ_3_ reach their respective troughs sequentially before reaching to peak values. Following the trough of angle θ_1_, the rate of change of angle θ_2_ begins to decline. Similarly, after the trough of angle θ_2_, the rate of change of angle θ_3_ starts to decrease. Subsequently, after the trough of angle θ_3_, the rate of change of angle θ_1_ begins to decrease, while the rates of change for angles θ_2_ and θ_3_ start to rise.

From the goose neck motion trajectory analysis, the motion trajectories of the marker points were similar, the motion trend was basically the same, and the motion of the joints exhibited obvious transmissibility. In particular, the transverse motion amplitude of the goose neck key points L1, L2, L3, L4, and L5 decreased in turn, and the longitudinal motion amplitude increased in turn. When the target position was in Area 1 and Area 4, the overall goose neck was firstly moving downward and then forward. During the forward movement of the goose neck, the marker points L1, L2, and L3 of the anterior-middle segment moved further downward, and the movement heights of the marker points L4 and L5 of the posterior segment were basically unchanged. When the target position is in Area 2 and Area 5, the overall goose neck was firstly moved downward, then adjusted upward, and finally moved forward. During the forward movement of the goose neck, the movement heights of the marker points L1, L2, and L3 in the anterior-middle segment were basically unchanged, and the marker points L4 and L5 in the posterior segment moved further upward. When the target position was in Area 3 and Area 6, anterior segment marker point L1 movement height was basically unchanged, middle-posterior segment marker points L2, L3, L4, L5 forward movement amplitude decreased in turn, and upward movement amplitude increased in turn.

To summarize, the goose neck movement had continuity and transmissibility, and the overall posture of the goose neck was adjusted through the synergistic movements of the anterior, middle, and posterior segments to complete the movement toward the target positions. Among them, the anterior segment mostly carried out transverse movement, with the significant horizontal adjustment; the middle segment combined transverse and longitudinal movement, with similar movement ability in all directions; and the posterior segment mostly carried out longitudinal movement, with a significant height adjustment.

This research investigated the muscle distribution characteristics in sagittal plane, coronal plane and transverse plane, researched the joint rotation characteristics and partition motion law, and revealed the mechanism of goose neck motion in narrow space. The next step will construct obstacle avoidance posture curve and analysis based on goose neck motion posture in narrow space to establish the bionic obstacle avoidance trajectory of robotic arms in narrow space and experimentally verify the reasonableness and superiority of the trajectory design. At present, the mechanism of the synergistic motion of the anterior, middle, and posterior segments of the goose neck based on the overall motion postures of the goose neck was analyzed only combined the high-speed camera motion data of the goose neck. The goose neck is a rigid-flexible coupled complex structure as a whole, and the bone-muscle synergy of the goose neck motion in the narrow space is still needed to be explored, which will provide theoretical support for the combination of robotic arms control strategies and structural design and further improve the flexibility and load capacity of the bionic robotic arms.

## 5 Conclusions

In this article, the goose neck was used as the test object to research the movement mechanism in different target areas in a narrow space. Firstly, goose neck MRI image data was collected. It was found that there are longitudinal muscles in both sagittal plane and coronal plane connecting the anterior and terminal parts of the goose neck, which controlled the lateral bending and rotational movements of the goose neck. The muscle areas in the transverse plane of the goose neck were different, the anterior segment was dominated by ventral flexion movement, so the ventral muscle area was larger; there was no significant difference in the area of the ventral and dorsal muscles in the middle segment; and the posterior segment was dominated by dorsiflexion movement, so the dorsal muscle area was larger.

Then, the motion data of the goose neck in the narrow space was collected by a high-speed camera system to analyze the goose neck motion:

1) The optimum value and the range of variation of the motion joint angle of the goose neck were analyzed, it was found that the anterior segment exhibited the greatest adjustment capability, followed by the middle segment, with the posterior segment demonstrating relatively weaker adjustment ability. It indicated that the regulation of the anterior segment of the goose neck was stronger than that of the middle and posterior segments, and its flexibility was better than that of the middle and posterior segments.
2) The joint angles of goose neck movement were analyzed. It was found that the overall trend of change for each joint angle was similar, mostly decreased first and then increased. In terms of the rate of change of the angles, mostly when the anterior segment joint angle θ_1_ reached the trough, the rate of change of the middle segment joint angle θ_2_ and the posterior segment joint angle θ_3_ decreased in turn. When the posterior segment joint angle θ_3_ reached the trough, the rate of change of the anterior segment joint angle θ_1_ began to decrease, and the rate of change of the middle segment joint angle θ_2_ and the posterior segment joint angle θ_3_ began to increase.
3) The motion trajectory of the goose neck was analyzed. It was found that the motion trajectories of each marker point were similar, the motion trend was basically the same. The motion travels of the marker points L1, L2, L3, L4, and L5 decreased in turn, and the motion of the joints exhibited obvious transmissibility. From the motion process, the overall goose neck was first height-adjusted, and when the height of the head was nearly the same as that of the target position, the horizontal movement was performed, and finally reached the target position.

The research results showed that the goose neck has excellent motion flexibility in narrow space and excellent obstacle avoidance effect. Its muscle distribution characteristics, joint rotation characteristics and partition motion law provided the important theoretical basis for designing the high flexibility and adaptability bionic robotic arm. In addition, the feasibility of the YOLOv7-pose recognition network for goose neck motion pose recognition was verified to provide a new method for animal behavior research.

## Data Availability

The original contributions presented in the study are included in the article/supplementary material, further inquiries can be directed to the corresponding author.
